# Effective and Elaborative Induction Program for Mitigating Myths and Misconceptions Linked to Hematopoietic Stem Cell Transplantation in a Resource Limited Setting

**DOI:** 10.1007/s12288-023-01634-5

**Published:** 2023-03-16

**Authors:** Safaa A. A. Khaled, Mahmoud M. Elzembely, Asmaa M. A. Soliman, Nahed Shwakat, Nashwa Rafaat, Mohamed A. Malek, Esmat S. Abdelmageed

**Affiliations:** 1 Department of Internal Medicine-Clinical Hematology Unit, Assiut University Hospital, Assiut, Egypt; 2 Unit of Bone Marrow Transplantation, South Egypt Cancer Institute, Faculty of Medicine, Assiut University, Assiut, Egypt; 3 Department of Pediatric Oncology, South Egypt Cancer Institute, Faculty of Medicine, Assiut University, Assiut, Egypt; 4 Department of Public Health and Community Medicine, Faculty of Medicine, Assiut University, Assiut, Egypt; 5 Department of Nursing Administration, Faculty of Nursing, Assiut University, Assiut, Egypt; 6 Department of Pharmacology, Faculty of Medicine, Assiut University, Assiut, Egypt; 7 Faculty of Medicine, Assiut University, Assiut, Egypt; 8 Department of Medical Surgical Nursing, Faculty of Nursing, Assiut University, Assiut, Egypt

**Keywords:** Myths, HSCT, Resource limited, Misconceptions

## Abstract

Since the first transplant in 1957 and hematopoietic stem cell transplantation (HSCT) is the curative modality for numerous hematological disorders. Nevertheless, it is not available for all patients. Besides unavailability of matched donors a lot of factors could hinder HSCT in a resource limited setting, as financial and administrative factors. In our daily practice we noticed other factors that hinder HSCT in our center, the common myths and misconceptions about HSCT and donation. This quasi-experimental study assessed, for the first time, common myths and misconceptions about HSCT among 218 medical and nursing students before and after an interventional educational program. The study tool was an investigators' developed self-administered questionnaire. Participants' male to female ratio was 1:2.5, and FAS was middle in 52.7%. Pretest high myths scores were reported in 53.4% and 90% of medical and nursing students that was reduced to 0% and 4% post-test, respectively. Pretest, 26.3% and 7% of medical and nursing students welling to donate HSC, that increased to 66% and 39% post-test, respectively. Rural residency, low and middle FAS associated with higher myths scores. Myths score is an independent effector of willingness to donate HSC among participants. In conclusion medical/nursing students had significant myths and misconceptions about HSCT that was corrected with the educational program. Thus, wide based educational programs about HSCT are mandatory to correct myths and augment HSC donation.

www.clinicaltrrial.gov: clinical trial ID NCT05151406.

**Supplementary Information:**

The online version contains supplementary material available at 10.1007/s12288-023-01634-5.

## Introduction

HSCT is currently used to treat a number of blood disorders [1]. The first transplant was performed by Thomas et al., and published in the New England Journal of Medicine [2]. Advancement of HSCT techniques and prevention of post procedure complications, prolonged the event free survival of HSCT recipients, thus expanded its use [3, 4]. Nevertheless, still many factors limit HSCT worldwide, particularly in limited resource regions. Those include unavailability of matched donors, high cost, rarity of specialized centers and health care professionals. Stem cell and cord blood banking helped to solve the donor unavailability in many centers [5, 6].

HSCT is a complex procedure requiring comprehensive education of patients, donors, and caregivers. A positive knowledge about HSCT among healthcare providers could increase unrelated donors. Health care professionals should try to ensure that potential donors have the right knowledge about the value and safety of the procedures and methods of donation and transplantation [7].

It was reported that lack of knowledge about HSCT among medical students may interfere with participation in the bone marrow registry. Therefore, educational programs can improve knowledge gap for the next generation of healthcare providers, thus enhance recruitment and retention of donor populations [8].

Myths about bone marrow donation are prevalent and must be dispelled to augment increase donation. Wide-spread barriers exist related to inaccurate perception of donation processes. These widely held beliefs prevent people from expressing willingness to donate. College students are an important target. So, educational efforts, by overcoming these perceived barriers, can increase HSC donation [9].

In Egypt HSCT started on 1989. On 2020, Egypt has 15 transplant centers with a transplant rate of 8.4 per million compared to 36–40 per million in western countries. Mahmoud et al. reported 30 years' experience of HSCT in Egypt with 60% of the performed transplants were allogeneic. However, still, a lot of challenges face HSCT in Egypt including, lack of HLA registry, lack of matched unrelated donors, lack of cord blood banking, limited haplo-identical program and socioeconomic factors. In Egypt we don't have local donor registry so we depend mainly on siblings [10, 11].

In 2016, the first HSCT center in Upper Egypt was established at South Egypt Cancer Institute (SECI), since then many cases were transplanted with good response. SECI is a big tertiary health center that serves millions of people who lived at Upper Egypt and distributed over 10-Governorates and their related cities and villages. In our daily practice we noticed a big challenge that could face HSCT and HSC donation in our center, the common myths and misconceptions about HSCT and donation. The source of these myths and misconceptions could be the TV shows, dramas and movies those represented HSCT as something scary and overstated HSC donation, particularly donation of bone marrow. Moreover, they represented post-transplant complications and /or post-donation discomfort in a dramatic manner. Owing to the spectacular effect of mass-media on shaping subjects' knowledge, thoughts and believes, these myths and misconceptions were common even among those highly educated personnel [12]. This motivated us to carry on this study in a trial to assess the size of the problem and establish a good solution. Thus, the study focused on assessing myths and misconceptions about HSCT, and suggesting a cost-effective intervention to overcome this challenge, taking our center as an example for resource limited settings.

Myths and misconceptions about HSCT and /or donation among medical and nursing students preclude their participation in HSC donation and decrease their enthusiasm to educate and encourage other potential donors. Thus, our study targeted both medical and nursing students at our Institution.

## Subjects and Methods

### Study Objectives, Design, Settings and Hypothesis

#### Primary Study Objective

Assessment of myths and misconceptions about HSCT and the effect of an educational intervention among final year medical and nursing students.

#### Secondary Study Objective

Explore the intention and willingness to donate stem cell, before and after an educational program, among final year medical and nursing students.

#### Design and Settings

An interventional Quasi Experimental study design (pre/posttest) was used for this work that was conducted at medical and nursing Faculties at our institution during the period from October to December 2021.

#### Research Hypothesis

There is a certain degree of myths and misconceptions about HSCT among medical and nursing students. Those could be corrected by a targeted educational program. Moreover, the intervention could potentiate willingness for stem cells donation among the study participants. In addition, the study assumed baseline differences in the knowledge about HSCT among 5th year medical students and 4th year nursing students. This assumption was based on differences in their study courses, the assumed main source of their knowledge.

### Study Subjects

Two groups of students were included in the study, Group 1: 5^th^ year undergraduate medical students and Group 2: 4^th^ year undergraduate nursing students. Each group was randomly selected and both did not have prior exposure or training with regards to HSCT. Willingness to participate in the study was a prerequisite for subject's inclusion. History of chronic illness and / or blood-related diseases of the student or someone of his/her family members were exclusion criteria.

Both groups 1 and 2 assumed to have knowledge about HSCT from their study courses, also they represent a sector from the general population that could be involved in a HSCT team in their future career. Their course work, regarding HSCT, is mainly theoretical, but include rotations on hematology-oncology floors to a certain degree, with no rotations at HSCT units.

#### Calculation of Sample Size

According to Epi-info program version 6 with significance level of 95%, power of 80% and prevalence of respondents had a 76.4% average correct response rate regarding knowledge of donation process [8], the total required sample size was 277.

### Study Tools and Methods

#### Development of a Structured Questionnaire

An investigators' developed structured questionnaire was self-administered to the study participants, after taking their consent. The questions were adopted from the investigators' clinical practice and experience as most of them were frequently asked by their patients or their relatives. Other questions were developed by the researchers after national and international literature review. The tool was developed in English language as in appendix A then translated to Arabic language, participants' first language (appendix B). The later form was used in the study. The tool consisted of three parts:

##### Part I-Aimed to Collect Socio-Demographic Data of the Study Participants

Including age, sex, marital status, educational level, occupation, residence, and family wealth.

Family wealth was assessed with Family Affluence Scale (FAS). We used a three point ordinal scale, where FAS low (score = 0–2) indicates low affluence, FAS medium (score = 3–5) indicates middle affluence, and FAS high (score = 6–9) indicates high affluence [13].

##### Part II-Aimed to Assess Source of Participants' Information Regarding HSCT and Their Willingness for Donation of HSC

Including source of their knowledge, willingness for getting more knowledge, and willingness for donation.

##### Part III-Aimed to Explore Myths and Misconceptions About HSCT Among the Study Participants

This was consisted of three domains; A: Myths and misconceptions about HSCT (10 questions), B: Myths and Misconceptions about HSC donation (23 questions), and C: Myths and Misconceptions about umbilical cord blood stem cell preservation (13 questions).

#### Validation of the Study Tool

Content validity and reliability of the study tool was established by a panel of seven experts (3 from BMT staff, 2 from Public Health staff, and 2 from Medical Surgical Nursing staff) who reviewed the study tools for clarity, relevance, simplicity, comprehensiveness, and applicability. Minor modifications were required. Then, the final form of the tool was designed and tested for reliability by using Cronbach's alpha that was calculated to the total score 46 value = 0.898.

#### Pilot Study

A pilot study was conducted on 10% of the study sample, to ensure clarity, examine applicability, and identify difficulties of the tools, also to determine the needed time to answer the questions. This group of participants was asked to answer both the English and Arabic versions of the study tool. Results of the pilot study revealed that the average time needed to complete the questionnaire was approximately 20 min. Based on the pilot study minor changes and modifications were applied to the study tool, so the sample piloted was excluded from the actual study sample.

#### The Educational Intervention

An educational program was developed by the researchers based on review of the relevant literature and textbooks to provide the study sample with the needed knowledge about HSCT in a trial to correct their myths and misconceptions about this type of treatment. The educational leaflet included three theoretical parts: First part included knowledge about HSCT as description of HSCT, definition, types, indications, contraindications, and how HSCT is performed. Second part included knowledge about HSC donation as Peripheral Blood Stem Cell donation (PBSCD), Bone marrow collection, and contraindications of donation. Third part included knowledge about stem cell banking as description of umbilical cord blood, reasons to store umbilical cord blood, the process of its collection, and method of preservation. This was in addition to educational lectures and sessions developed and presented by the researchers.

#### Procedure

The study proceeded in three phases.

##### Assessment Phase (Pretest)

The researchers met the selected studied sample; each one of them was fully informed with the purpose and nature of the study and their agreement was obtained. Base line data of the myths and misconceptions about HSCT among the study participants were collected using the validated study tool (parts I, II, and III).

##### Implementation Phase (Intervention)

- The educational program was delivered by the researchers; each participant received an educational leaflet and included in educational sessions (3-sessions) that included a group of students. Each session lasted about 50-mimutes.
- During the session extensive literature review that included pictures and guidance about HSCT to correct participants' myths and misconceptions in clear Arabic language, to help them retain the learned material.
- A brief review was elicited from the study sample to assess their understanding, then the researchers clarified any points that they did not understand.

##### Evaluation Phase (Posttest)

In this phase, the studied sample was reassessed at the end of the educational session using the validated study tool (parts II and part III), to evaluate the effectiveness of the educational program on correcting their myths and misconceptions about HSCT.

#### Data Collection and Scoring System

Participants' answers of the pre and post-tests were collected. A total misconception score for HSCT was calculated. The misconceptions score measured on 0–1 scale for each item, giving one point for each correct response, whereas incorrect or unknown responses received zero points (the total for all items was 46 (10 items for bone marrow transplantation, 23 items for bone marrow donation & 13 items for stem cell banking). The overall misconceptions score was dichotomized as High level of Myths if the correct answers < 50%, Moderate level of Myths 50%—< 70% and Low level of Myths ≥ 70%, by cutoff level of 23, which was the median score of the distribution.

#### Statistical Analyses

Data entry and analysis were carried out using Statistical Package for Social Sciences (SPSS) version16. Descriptive statistics were represented in the form of frequencies, mean and SD. The X^2^ test and independent sample T-test were used to compare between the study groups and the paired sample T-test to compare pre and post-test for each group. The Pearson's correlation coefficient and Chi-square test were used to assess association between sociodemographic characteristics and total score of corrected answers of medical /nursing faculty students/ and willingness to donate HSC. Values were considered significant when P values less than 0.05.

## Results

### Sociodemographic Characteristics of the Study Participants (Part I)

**Fig. 1:**
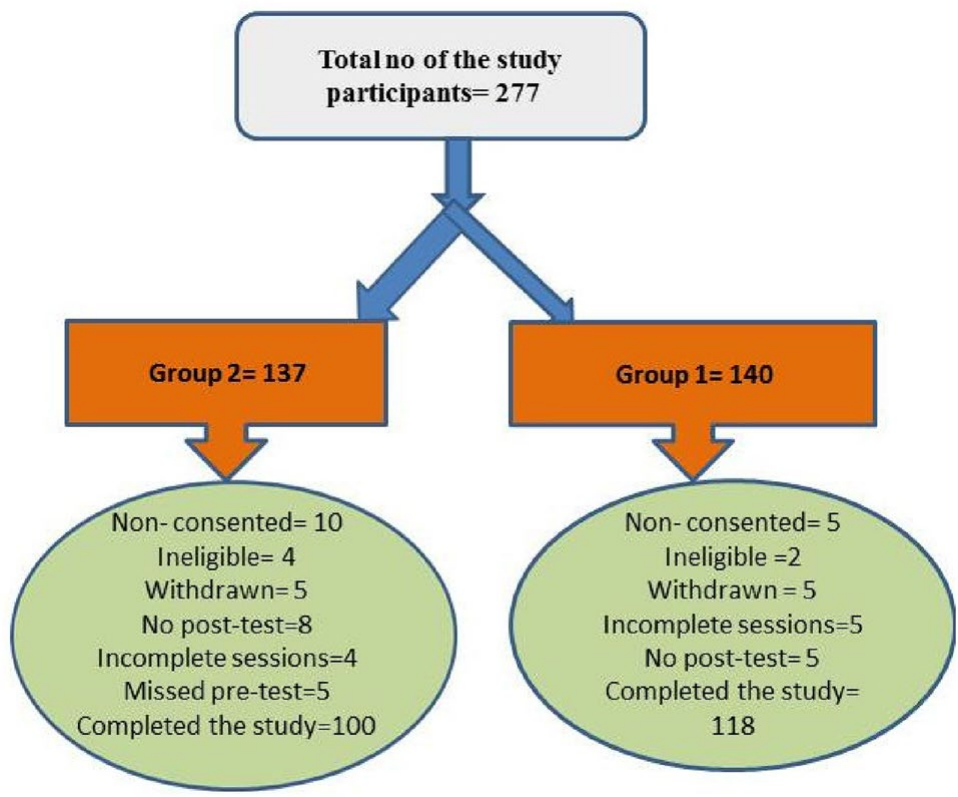
Flowchart of the study participants

A total of 277 student were enrolled in the study however only 218 completed the study (group 1 = 118 and group 2 = 100) due to ineligibility, withdrawals, non –consenting, and missing. Figure 1 illustrated flowchart of the study participants. All of them were in age range 22–23 years old, Table 1 showed sociodemographic characteristics of the study participants. It revealed no statistically significant differences between the two groups as regard gender, marital status and residence. However significant difference among both groups was noted when considering FAS where moderate and high FAS scores were reported among 52.5% &47.2% and 17.8% & 5% of medical and nursing students, respectively (P < 0.000). Notably three fourths of the study participants were females. with a male to female ratio 1:2.6. 11% of the whole studied sample were married, 58.7% of participants lived in rural areas, and only 11.9% of them had high FAS scale.

**Table 1:** Comparison of sociodemographic characteristics among the study groups, *X*^*2*^ test (total n = 218)

Personal data	Medicine (n = 118)		Nursing (n = 100)		Total (n = 218)		*P*-value
	No	%	No	%	No	%	
Sex							
Male	36	30.5	25	25.0	61	28.0	0.367
Female	82	69.5	75	75.0	157	72.0	
Marital status							
Single	106	89.8	88	88.0	194	89.0	0.667
Married	12	10.2	12	12.0	24	11.0	
Governorate							
Assiut	76	64.4	90	90.0	166	76.1	
Sohag	19	16.1	0	0.0	19	8.7	
Luxor	5	4.2	0	0.0	5	2.3	
Qena	1	0.8	0	0.0	1	0.5	–
Aswan	1	0.8	0	0.0	1	0.5	
Al-Minia	15	12.7	1	1.0	16	7.3	
Al-Wadi Al-Gadeed	1	0.8	9	9.0	10	4.6	
Residence							
Rural	63	53.4	65	65.0	128	58.7	0.083
Urban	55	46.6	35	35.0	90	41.3	
Family affluence scale							
Low	35	29.7	54	54.0	89	40.8	
Middle	62	52.5	41	41.0	103	47.2	0.000**
High	21	17.8	5	5.0	26	11.9	
Source of information							
Educational materials	36	30.5	14	14.0	50	22.9	
Health team	15	12.7	1	1.0	16	7.3	0.000**
Internet	16	13.6	14	14.0	30	13.8	
Faculty	51	43.2	71	71.0	122	56.0	
Need knowledge about HSCT							
Yes	118	100.0	93	93.0	211	96.8	0.004**
No	0	0.0	7	7.0	7	3.2	

**Highly significant

### Source of Participants' Knowledge Regarding HSCT (Part II)

Table 1 showed that in about half of medical and nursing students (43.2% & 56%), respectively, the source of their information about HSCT is the faculty. All medical students and the majority of nursing students have a strong well to gain further knowledge about HSCT with a statistically significant difference among both the study groups.

### Myths and Misconceptions About HSCT Among the Study Participants (Part III Domains A, B and C) and the Impact of the Educational Intervention (Pre and Post-Tests)

Table 2. showed that the highest myth and misconception as regard HSCT among medical and nursing students was "surgeons perform bone marrow transplantation operations" (88.1% & 98%) respectively (pre-test). This was decreased to (12.7% & 35%) respectively (post-test).

**Table 2:** Myths and misconceptions about HSCT among the study groups pre/post intervention (total n = 218)

Myths and misconceptions	Medical				*P*-value	Nursing				*P*-value	Total				*P*-value
	Pre-test		Post-test			Pre-test		Post-test			Pre-test		Post-test		
	No	%	No	%		No	%	No	%		No	%	No	%	
**A**-**Myths and misconceptions about HSCT**															
A1-There is only one type of HSCT	50	42.4	2	1.7	0.000*	63	63.0	9	9.0	0.000*	113	51.8	11	5.0	0.000*
A2-A stem cell transplant is a surgical procedure that requires the patient to enter the operating room and be given general anesthesia	80	67.8	63	53.4	0.024*	88	88.0	10	10.0	0.000*	168	77.1	73	33.5	0.000*
A3-Surgeons perform bone marrow transplantation operations	104	88.1	15	12.7	0.000*	98	98.0	35	35.0	0.000*	202	92.7	140	64.2	0.000*
A4-HSCT is used in treating cancer only	21	17.8	1	0.8	0.000*	40	40.0	2	2.0	0.000*	61	28.0	3	1.4	0.000*
A5-After the recipient discharged from the hospital, periodic follow-up must be done at regular periods in the hospital	8	6.8	3	2.5	0.123	21	21.0	8	8.0	0.009*	29	13.3	33	15.1	0.583
A6-Visiting the recipient is prohibited after the stem cells transfer for a period specified by the doctors	24	20.3	23	19.5	0.871	43	43.0	10	10.0	0.000*	67	30.7	60	27.5	0.461
A7-The cure rate is very poor after HSCT	37	31.4	20	16.9	0.010*	57	57.0	8	8.0	0.000*	94	43.1	28	12.8	0.000*
A8-The recipient is kept in the hospital for long periods up to years	43	36.4	3	2.5	0.000*	43	43.0	8	8.0	0.000*	86	39.4	11	5.0	0.000*
A9-The HSCT is a simple procedure with no complications	52	44.1	37	31.4	0.044*	34	34.0	41	41.0	0.307	86	39.4	78	35.8	0.429
A10-There is a HSCT center in your area	99	83.9	11	9.3	0.000*	97	97.0	16	16.0	0.000*	196	89.9	27	6.2	0.000*
**B**-**Myths and Misconceptions about bone marrow donation**															
**B1**-I do not need to be a HSC donor as long as I am not sick	34	28.8	30	25.4	0.558	49	49.0	9	9.0	0.000*	83	38.1	39	17.9	0.000*
**B2**-Bone marrow donation is done by removing part of the bone and extracting bone marrow from it	65	55.1	19	16.1	0.000*	78	78.0	13	13.0	0.000*	143	65.6	32	14.7	0.000*
**B3**-Donating bone marrow may be associated with paralysis after donation	66	55.9	3	2.5	0.000*	84	84.0	18	18.0	0.000*	150	68.8	21	9.6	0.000*
**B4**-Donating bone marrow affects the fertility of men or women	60	50.8	0	0.0	0.000*	78	78.0	17	17.0	0.000*	138	63.3	17	7.8	0.000*
**B5**-Donating bone marrow makes the patient vulnerable to cancer	41	34.7	12	10.2	0.000*	75	75.0	5	5.0	0.000*	116	53.2	17	7.8	0.000*
**B6**-Donating HSC is not necessarily from a brother, but is suitable for the father or mother or any relative	31	26.3	19	16.1	0.056	59	59.0	15	15.0	0.000*	90	41.3	63	28.9	0.007*
**B7**-Children are not suitable for stem cells donation	64	54.2	4	3.4	0.000*	83	83.0	82	82.0	0.852	147	67.4	150	68.8	0.758
**B8**-Donating stem cells affect the growth of children	66	55.9	9	7.6	0.000*	81	81.0	44	44.0	0.000*	147	67.4	53	24.3	0.000*
**B9**-A blood transfusion may be given after donating bone marrow	51	43.2	80	67.8	0.000*	61	61.0	60	60.0	0.885	112	51.4	140	64.2	0.007*
**B10**—Donation from a man to a woman is not suitable, or vice versa	35	29.7	14	11.9	0.001*	61	61.0	17	17.0	0.000*	96	44.0	31	14.2	0.000*
**B11**-The donor may be admitted to the hospital after donation	41	34.7	76	64.4	0.000*	38	38.0	75	75.0	0.000*	79	36.2	164	75.2	0.000*
**B12**-The age of the donor does not matter	76	64.4	15	12.7	0.000*	46	46.0	13	13.0	0.000*	122	56.0	28	12.8	0.000*
**B13**-Donating HSC takes a long time, so it is a waste of time	36	30.5	37	31.4	0.888	42	42.0	9	9.0	0.000*	78	35.8	46	21.1	0.001*
**B14**-If you are a woman, the donor must also be a woman	23	19.5	17	14.4	0.298	65	65.0	10	10.0	0.000*	88	40.4	27	12.4	0.000*
**B15**-Surgery is the only way to donate HSC	82	69.5	5	4.2	0.000*	87	87.0	2	2.0	0.000*	169	77.5	7	3.2	0.000*
**B16**-Donation is only from relatives	32	27.1	2	1.7	0.000*	75	75.0	15	15.0	0.000*	107	49.1	17	7.8	0.000*
**B17**-If I am a woman and the donor is a man, I will have masculine characteristics	45	38.1	1	0.8	0.000*	60	60.0	11	11.0	0.000*	105	48.2	12	5.5	0.000*
**B18**-Donating stem cells is dangerous and weakens the donor	77	65.3	19	16.1	0.000*	77	77.0	7	7.0	0.000*	154	70.6	26	11.9	0.000*
**B19**-HSC donation is really painful	61	51.7	39	33.1	0.004*	89	89.0	17	17.0	0.000*	150	68.8	56	25.7	0.000*
**B20**-Donation of stem cells includes a long recovery period	88	74.6	58	49.2	0.000*	92	92.0	40	40.0	0.000*	180	82.6	98	45.0	0.000*
**B21**-Registration to donate bone marrow requires a blood test	74	62.7	101	85.6	0.000*	30	30.0	13	13.0	0.003*	104	47.7	114	52.3	0.338
**B22**-The bone marrow is taken from the spine	82	69.5	34	28.8	0.000*	90	90.0	23	23.0	0.000*	172	78.9	57	26.1	0.000*
**B23**-Donating HSC is very expensive	45	38.1	38	32.2	0.340	53	53.0	89	89.0	0.000*	98	45.0	127	58.3	0.005*
**C: Myths and Misconceptions about umblical cord blood stem cell preservation**															
**C1**-If someone in my family needs a cord blood stem cell transplant, it can only be done if I donate cord blood to my baby	86	72.9	58	49.2	0.000*	96	96.0	65	65.0	0.000*	182	83.5	123	56.4	0.000*
**C2**-If I preserved the cord blood for my first child, I do not need to preserve the cord blood for my second child	93	78.8	46	39.0	0.000*	88	88.0	39	39.0	0.000*	181	83.0	85	39.0	0.000*
**C3**-If I do not preserve my first child's stem cells, my second child's stem cells cannot be saved	96	81.4	19	16.1	0.000*	88	88.0	13	13.0	0.000*	184	84.4	32	14.7	0.000*
**C4**-Treatments by cord blood are limited to treating children	64	54.2	0	0.0	0.000*	85	85.0	10	10.0	0.000*	149	68.3	10	4.6	0.000*
**C5**-Umbilical cord blood transplantation is restricted to treating blood diseases only	54	45.8	14	11.9	0.000*	85	85.0	19	19.0	0.000*	139	63.8	33	15.1	0.000*
**C6**-Umbilical cord blood can be donated in any hospital	101	85.6	57	48.3	0.000*	69	69.0	60	60.0	0.184	170	78.0	117	53.7	0.000*
**C7**-A family expected to donate cord blood has time until birth to decide whether to donate	82	69.5	5	4.2	0.000*	94	94.0	87	87.0	0.091	130	59.6	134	61.5	0.695
**C8-**Collecting umbilical cord blood could harm my baby	31	26.3	18	15.3	0.037*	83	83.0	3	3.0	0.000*	114	52.3	21	9.6	0.000*
**C9**-Cord blood is worthless medical waste	61	51.7	34	28.8	0.000*	61	61.0	39	39.0	0.002*	122	56.0	73	33.5	0.000*
**C10**-Stem cell collection is a risky medical procedure	35	29.7	5	4.2	0.000*	92	92.0	18	18.0	0.000*	127	58.3	23	10.6	0.000*
**C11-**Stem cell preservation is only for families with a history of cancer	32	27.1	14	11.9	0.003*	75	75.0	10	10.0	0.000*	107	49.1	24	11.0	0.000*
**C12-**Umbilical cord blood collection can affect delivery and draw blood from our baby	77	65.3	44	37.3	0.000*	85	85.0	10	10.0	0.000*	162	74.3	54	24.8	0.000*
**C13-**Preserved umbilical cord blood has a "limited shelf life."	20	16.9	21	17.8	0.864	99	99.0	55	55.0	0.000*	119	54.6	76	34.9	0.000*

The highest myth and misconception as regard HSC donation among medical and nursing students was "the bone marrow is taken from the spine"(69.5% & 90%), respectively (pre-test) this was reduced to (28.8% &23%) respectively (post-test) with a statistically significant difference.

The highest myth and misconception as regard umbilical cord blood stem cell preservation among medical and nursing students was" if I do not preserve my first child's stem cells, my second child's stem cells cannot be saved" (81.4% & 88%), respectively (pre-test). This was converted to (16.1% &13%) respectively (post-test) with statistically significant difference.

Strikingly, most of medical /nursing students did not realize that there is a HSCT center in their locality; this was corrected after the intervention.

### Total Scores of Myths and Misconceptions About HSCT Among the Study Groups and the Impact of the Educational Intervention (Pre and Post-tests)

**Fig. 2:**
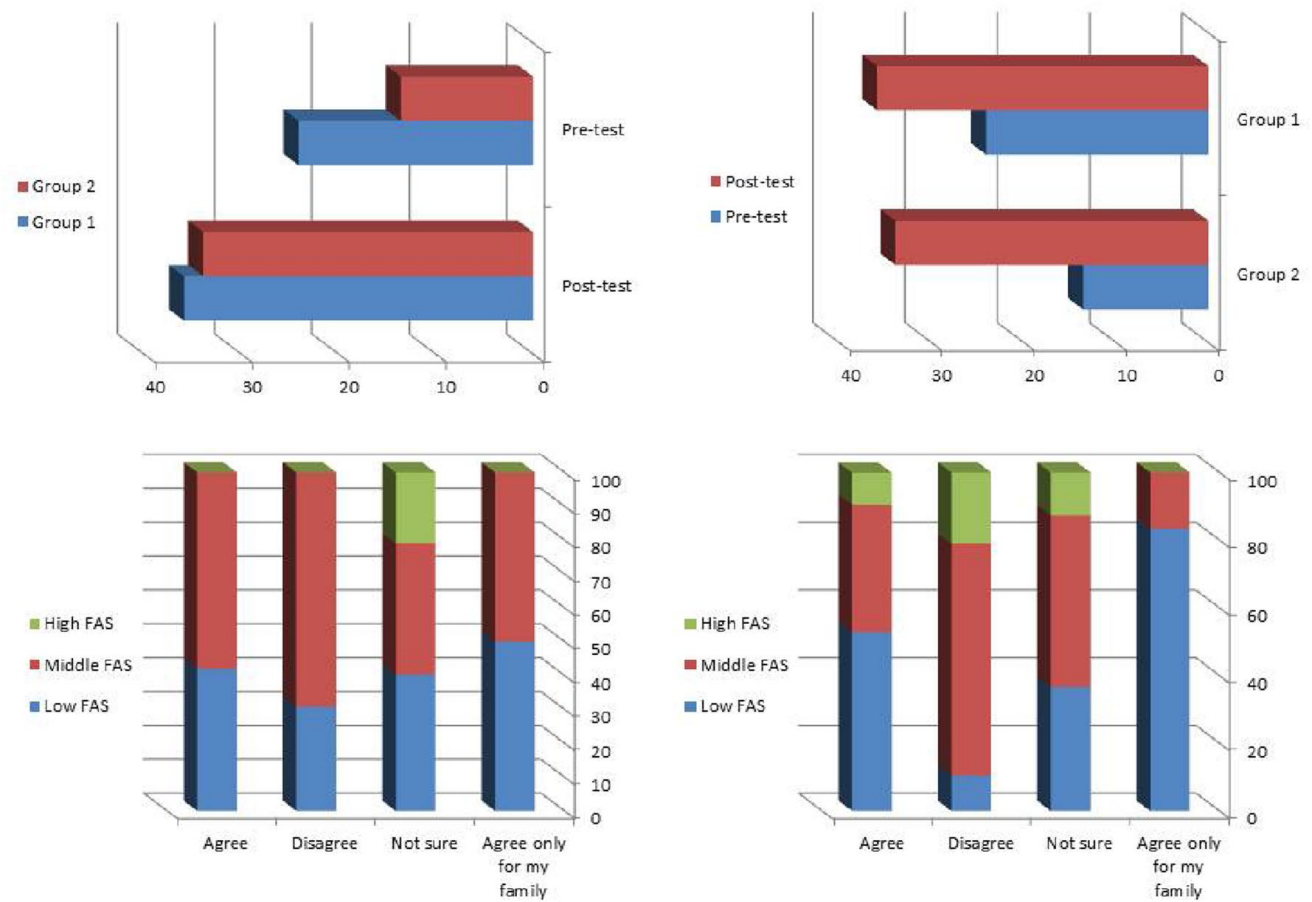
Differences of myths and misconceptions mean scores (upper panel right and left) among the study groups, and between pre-and post-tests within the same group, respectively. Lower panel right and left, association between FAS of the study participants and their willingness to donate HSC before and after the intervention, respectively

Figure 2 (upper panel right) and supplementary tables S1-4 showed comparison of baseline and post-test mean scores of myths and misconceptions about HSCT between groups 1 and 2, there were statistically significant differences between the two groups whether before or after the intervention. Figure 2 (upper panel left) illustrated mean scores within each group before and after the intervention.

Table 3 showed that about half (53.4%) of medical students and 90% of nursing students had high level of myths and misconceptions (pre-test), while in post-test the high level of myths in medical students was zero and 1.8% in nursing students with statistically significant difference between groups.

**Table 3:** Comparison of total scores of myths and misconceptions about HSCT, and willingness to donate stem cells among the study groups before and after the intervention (total no = 218)

Total score	Medicine (n = 118)		Nursing (n = 100)		Total (n = 218)		*P*-value^1^
	No	%	No	%	No	%	
**Myths and misconceptions scores**							
*Pre-test*							
High level of Myths	63	53.4	90	90.0	153	70.2	
Moderate level of Myths	34	28.8	9	9.0	43	19.7	0.000**
Low level of Myths	21	17.8	1	1.0	22	10.1	
*Post-test*							
High level of Myths	0	0.0	4	4.0	4	1.8	
Moderate level of Myths	16	13.6	22	22.0	38	17.4	0.019*
Low level of Myths	102	86.4	74	74.0	176	80.7	
*P*-value^2^	0.000**		0.000**		0.000**		
**Willingness to donate stem cells**							
*Pre-test*							
Agree	31	26.3	7	7.0	38	17.4	
Disagree	17	14.4	9	9.0	26	11.9	0.000**
Not sure	63	53.4	61	61.0	124	56.9	
Agree only to my family	7	5.9	23	23.0	30	13.8	
*Post-test:*							
Agree	78	66.1	39	39.0	53	24.3	
Disagree	10	8.5	2	2.0	19	8.7	0.000**
Not sure	29	24.6	50	50.0	134	61.5	
Agree only to my family	1	0.8	9	9.0	12	5.5	
*P*-value^2^	0.000**		0.000**		0.009**		

N.B. P1: differences between groups, P2: differences of pre and post-test scores within the same group. *Significant, **highly significant

### Baseline Willingness to Donate Stem Cells Among the Study Groups and Impact of the Educational Intervention (Pre and Post-Tests)

Table 3 showed willingness of both medical and nursing students to donate stem cells before and after the intervention. 26.4% & 7% of medical and nursing students willing to donate before the intervention, this was increased to 66.1% and 39% after the educational program, respectively, with significant differences between groups.

### Factors Affecting Myths and Misconceptions About HSCT Among the Study Participants

Table (S5) showed the association between sociodemographic characteristics and the total score of corrected answers among the study participants. It revealed no significant effect of gender, marital status or FAS scale. However, there was increase in mean scores of corrected answers as regard type of faculty (pre/posttest) and residence (post – test) with statistically significant association.

### Factors Affecting Willingness to Donate Stem Cells Among the Study Participants Before and After the Educational Intervention (Pre and Post-tests)

Table 4 showed the association between willingness to donate HSC and sociodemographic characteristics and myths and misconceptions scores among the study participants (pre/post-test). Before the intervention there was no significant effect of gender on willingness to donate HSC that became highly significant after the intervention. Notably, there was a significant effect of residency, before the intervention where nearly two thirds of those who agree to donate HSC reside in rural areas.

**Table 4:** Association between sociodemographic characteristics, the total score of myths and willingness to donate HSC among the study participants (before and after the intervention)

Variables	Willingness to donate HSC							
	Pre-test (N (%))				Post-test (N (%))			
	Agree	Disagree	Not-sure	Agree only for my family	Agree	Disagree	Not-sure	Agree only for my family
**Myths score**								
High	29 (76.3)	25 (96.2)	77 (62.1)	23 (76.7)	0 (0)	0 (0)	3 (2.2)	1 (8.3)
Moderate	5 (13.2)	1 (3.8)	29 (23.4)	5 (16.7)	2 (3.8)	19 (100)	16 (11.9)	1 (8.3)
Low	4 (10.5)	0 (0)	18 (14.5)	2 (6.7)	51 (96.2)	0 (3.8)	115 (85.8)	10 (83.3)
*P*-value	0.029*				0.000**			
**Sex**								
Male	13 (34.2)	9 (34.6)	35 (28.2)	4 (13.3)	19 (31.1)	12 (19.7)	29 (47.5)	1 (1.6)
Female	25 (65.8)	17 (65.4)	89 (71.8)	26 (86.7)	34 (21.7)	7 (4.5)	105 (66.9)	11 (7)
*P*-value	0.213				0.000**			
**Marital status**								
Single	37 (97.1)	13 (50)	116 (93.5)	2 (93.3)	48 (90.6)	18 (94.7)	117 (87.3)	11 (91.7)
Married	1 (2.6)	13 (50)	8 (6.5)	2 (6.7)	5 (9.4)	1 (5.3)	17 (12.7)	1 (8.3)
*P*-value	0.000**				0.742			
**Governorate**								
Assiut	20 (52.6)	26 (100)	96 (77.4)	24 (80)	49 (92.5)	17 (89.5)	90 (67.2)	10 (83.3)
Other governorates	18 (47.4)	0 (0)	28 (22.6)	6 (20)	4 (7.5)	2 (10.5)	44 (32.8)	2 (16.7)
*P*-value	0.000**				0.001**			
**Residence**								
Rural	24 (63.2)	22 (84.6)	65 (52.4)	17 (56.7)	22 (41.5)	10 (52.6)	52 (38.8)	6 (50)
Urban	14 (36.8)	4 (15.4)	59 (47.6)	13 (43.3)	25 (15.9)	9 (47.4)	82 (61.2)	6 (50)
*P*-value	0.022*				0.631			

N.B. HSC = hematopoietic stem cell, *Significant, **highly significant

Considering the association between myths and misconceptions score of the studied sample and theirs well to donate HSC, before the intervention, nearly 96% who disagree to donate had high and the remaining 4% had moderate scores. On the contrary none of those who disagree to donate had low scores. After the intervention 96.2 and 3.8 who agree to donate HSC had low and moderate scores, respectively, meanwhile 100% who disagree had moderate scores.

Figure 2 (lower panel right and left), and suppl. Tables S6 and S7 showed the association between willingness to donate stem cells, pre/post-test respectively, and FAS scale, of the study participants. They, revealed that, before the intervention, participants whose their FAS is high were mainly not sure whether to donate or not and none of those who agree to donate HSC had high FAS scale. On the contrary, the vast majority of those with middle FAS willing to donate stem cells. After the intervention 9.4% of those who agree to donate HSC with high FAS.

## Discussion

HSCT whether allogeneic or autologous, is the hope for many patients with hematological disorders [14]. Recently we started HSCT in our newly developed center, since then many cases were transplanted with good response, however still the number of transplanted cases did not match the number of patients eligible for transplantation. This was in part due to high cost of the procedure and the long administrative and technical pre-transplant measures. Nevertheless, the most important challenge that faces HSCT at our institution is the unavailability of matched related or unrelated donors. Even patients' relatives hesitated or totally refused to go through matching tests and donate HSC for their patient. In our daily practice We reported a lot of myths and misconceptions about HSCT and donation. and we assume that believes could play an important role in the problem. Thus, we conducted this study in a trial to identify these myths and misconceptions, their impact on HSCT and donation behavior and find out a cost-effective method to correct them. To do so, we recruited 218-medical and nursing final year students, after their consent and administrative approval of the Vice Deans of students' affairs. Their myths and misconceptions about HSCT and donation were assessed before and after an educational program (pre/post-test). Their willingness to be potential HSC donors was assessed, too.

Results of the study showed that nearly three fourths of the study participants were female students. Besides the female predominance of nursing college, this could be explained by the results of Stefan who concluded that males have less motivation to have health related information or engaged in health surveys [15]. Nearly half of the current study participants reside in rural areas and have Middle FAS. These results represented the real geographic distribution and sociodemographic characteristics of the studied population.

This study reported that the main source of medical/nursing students' information about HSCT is their study courses; this was albeit similar to what was reported in Narayanan et al. study, 68% [8]. In other studies, the main source of information was the internet [16, 17]. An astonishing finding of the current study pretest was that most participants do not know that there is a HSCT center in their locality. This finding highlighted the ultimate need for wide base educational programs about HSCT even among medical and nursing students.

Regarding the myths and misconceptions about HSCT among the studied sample, the highest percentage of both medical and nursing students had high levels of myths and misconceptions before implementation of the educational program (pre-test), this may be due to the fact that HSCT is a new advanced trend in health care. Furthermore, medical and nursing curriculums stayed deficient in this issue. This result was supported by Kim and Ahn who assessed nursing student knowledge and attitude regarding HSCT in Korea [18]. Also was comparable to that reported by Narayanan et al. [8], who studied medical students' knowledge and attitudes towards HSCT, although that 43% of their respondents were already on the United States national bone marrow registry program. However, results of this study were in contrast to Lai et al. who found high level of awareness to HSCT among medical students [19].

This study revealed that, In post-test after implementation of the educational program the highest percentage of both medical and nursing students had low levels of myths and misconceptions. From the researchers’ point of view this may be attributed to the effect of the educational program in improving knowledge of the students regarding HSCT, donation and banking. which helped in correcting myths and misconceptions among those students. Similarly, Kaya et al. reported that the knowledge and awareness of students in medical school improved after targeted education about HSCT [20]. Also, Kim and Shin showed a significant increase in knowledge of nursing students about HSCT [21].

This study reported higher incidence of myths and misconceptions about HSCT among nursing students, in those residing in rural areas and those with low or middle FAS. These differences persisted even after the intervention. The first finding could be explained by differences in study courses between nursing and medical students, and the last by unavailability of educational programs and other information sources.

The study goes further and assessed willingness for donating HSC, both groups (medical & nursing students) showed increase in their willingness to donate in post- than pre-test. This may be due to the positive contribution of the educational program in promoting students` knowledge about the benefit of HSCT and correcting their myths and misconceptions about requirements and hazards of donation. Thus, eliminated their relevant fears. In this regard Kim and Shin [21], Kwok et al. & McGlade & Pierscionek supported the current results as they found higher knowledge level leads to higher level of willingness and intention for donation. Also, Cebeci et al. stated that the healthcare provider’s education about HSCT helped giving correct knowledge to donors, leading to the desirable decisions for donations. They added, correct information about organ donation among nurses can promote donor’s willingness and help recipients and their families to enhance public awareness of donation [22–24].

Finally, the study reported that the main factors affecting participants' willingness to donate HSC were the total myths and misconceptions score followed by their FAS. Accordingly, we speculated that the educational programs, not only, can significantly correct myths and misconceptions about HSCT but also improve willingness for HSC donation.

## Conclusions and Recommendations

In conclusion this study reported a significant degree of myths and misconceptions about HSCT among medical and nursing students that were nearly corrected with an educational program. Moreover, the study proved that these myths and misconceptions could be independent factors that affect HSC donation behavior among students.

Based on these results we can say that to increase the ratio of donors-to-patients, not only more efforts to encourage donations needed, but we also need to provide educational programs for medical and nursing students. Both of them are the next generation of healthcare providers, to have correct knowledge and self-confidence about HSCT to fit this contemporary trend in health care. In addition, the wide range implementation of these programs on the population could enhance development of future stem cell registry and stem cell banking.

## Recommendations

### This Study Recommended

- Within the curriculum of medical and nursing colleges, it is necessary to include new innovations on health as HSCT to be included in more practical pattern.
- It is necessary to have the government’s policies and budget for cord blood stem cells bank and donor registry programs.
- Educational campaigns for college students and general population regarding HSCT and donation need to be developed as they are the future donors.
- Mass media educational programs about HSCT should be implemented to allow targeting a big sector of the population.

### Supplementary Information

Below is the link to the electronic supplementary material.

Supplementary file 1 (PDF 209 KB)

Supplementary file 2 (PDF 182 KB)

## Data Availability

The authors confirm that all data supporting the findings of this study are available within the article and the supplementary file.
