# Supplementary material for: Effective and Elaborative Induction Program for Mitigating Myths and Misconceptions Linked to Hematopoietic Stem Cell Transplantation in a Resource Limited Setting
Source: Indian J Hematol Blood Transfus. 2023 Mar 16;39(4):598–609. doi: 10.1007/s12288-023-01634-5 (PMC10542043; doi:10.1007/s12288-023-01634-5)
Supplement: Supplementary file 1 — (PDF 209 KB) [file 12288_2023_1634_MOESM1_ESM.pdf]

Appendix A

إستبيان لتقييم الأخطاء الشائعة والمفاهيم المغلوطة حول زراعة الخلاياً الجذعية الدمويةٍ

(زراعة النخاع)

التسلسل:

*** ما هو المصدر الرئيسي للمعلومات الخاصة بك؟**

1.المواد التعليمية بالمستشفى. 2.العاملين في المجال الطبي. 3. الدراما التليفزيونية

4. الانترنت 5. برامج التثقيف بالراديو والتليفزيون 6. صحف 7. مدرسة

8.كلية 9. مجلة 10. مؤتمر 11. اخري (يذكر)

*** هل ترغب في الحصول على معلومات حول زراعة الخلاياً الجذعية الدمويةٍ (زراعة النخاع)؟**

1. نعم 2. لا

*** هل ستتبرع بالخلايا الجذعية؟**

أ. بالتأكيد سوف أفعل. ب. لن أفعل أبدا.

ج. سوف أفكر في ذلك. د. بالتأكيد سوف أفعل إذا تم استخدامه فقط لعائلتي.

A- زراعة الخلاياً الجذعية الدموية:	نعم	لا
1- هناك نوع واحد فقط من زراعة الخلاياً الجذعية الدموية (زراعة النخاع).		
2- عمليه زراعة الخلايا الجذعية الدموية (زراعة النخاع) هي عمليه جراحيه تتطلب دخول المريض غرفة العمليات وتخديره تخدير كلي.		
3- يقوم الجراحون بعمل عمليات زراعة النخاع.		
4- تستخدم عملية زراعة النخاع في علاج السرطان فقط		
5- بعد خروج المتلقي من المستشفى يجب المتابعة الدورية علي فترات منتظمة بالمستشفى		
6- يتم منع الزيارة عن المتلقي بعد نقل الخلايا الجذعية لفتره يحددها الاطباء		
7- نسبة الشفاء ضعيفة جدا بعد عمليات زراعة الخلاياً الجذعية الدموية		
8- المتلقي يتم حجزه في المستشفى لفترات طويله تصل لسنوات		
9- عمليه زراعة الخلايا الجذعية الدموية (زراعة النخاع) عمليه بسيطة ولا يصحبها مضاعفات		
10- يوجد مركز لزراعة الخلايا الجذعية الدموية (زراعة النخاع) في منطقتك		

**B- التبرع بالخلايا الجذعية الدموية:**		
1- لا أحتاج أن أكون متبرع بالخلايا الجذعية الدموية طالما ليس لي مريض.		
2- التبرع بالخلايا الجذعية الدموية يتم من خلال ازالة جزء من العظام واستخلاص نخاع العظام منها		
3- التبرع بالخلايا الجذعية الدموية قد يكون مصحوبا بحدوث شلل بعد التبرع		
4- التبرع بالخلايا الجذعية الدموية يؤثر على خصوبة الرجل او المرأة		
5- التبرع بالخلايا الجذعية الدموية يجعل المريض عرضه للإصابة بالسرطان		
6- التبرع بالخلايا الجذعية الدموية ليس بالضرورة من الأخ ولكن يصلح من الاب او الام او اي قريب		
7- الأطفال لا يصلحون للتبرع بالخلايا الجذعية الدموية		
8- التبرع بالخلايا الجذعية الدموية يؤثر على نمو الاطفال		
9- قد يتم نقل دم بعد التبرع بالخلايا الجذعية الدموية		
10-لايصلح التبرع من رجل الي امراه او العكس		
11-قد يتم حجز المتبرع في المستشفي بعد التبرع		
12-عمر المتبرع لا يهم.		
13-التبرع بالخلايا الجذعية الدموية يستغرق وقتًا طويلاً لذا فهو مضيع للوقت.		
14-إذا كنت امرأة، فيجب ان يكون متبرعي امرأة أيضًا.		
15-الجراحة هي الطريقة الوحيدة للتبرع بالخلايا الجذعية الدموية.		
16-التبرع يكون فقط من الاقرباء.		
17-إذا كنت امرأة والمتبرع رجلًا، فسوف تظهر عندي سمات ذكورية.		
18-التبرع بالخلايا الجذعية الدموية أمر خطير ويضعف المتبرع.		
19-التبرع بالخلايا الجذعية الدموية مؤلم حقًا.		
20-يتضمن التبرع بالخلايا الجذعية الدموية فترة نقاهة طويلة.		
21-يتطلب التسجيل للتبرع بالخلايا الجذعية الدموية اختبار الدم.		
22-يتم أخذ الخلايا الجذعية الدموية من النخاع العظمي من العمود الفقري.		
23-التبرع بالخلايا الجذعية الدموية باهظ الثمن.		

		**C - حفظ الخلايا الجذعية من دم الحبل السري:**
		1- إذا كان شخص ما في عائلتي يحتاج إلى عملية زراعة خلايا جذعية من دم الحبل السري، فيمكن ذلك فقط إذا تبرعت بدم الحبل السري لطفلي.
		2- إذا قمت بحفظ دم الحبل السري لطفلي الأول، فانا لا احتاج إلى حفظ دم الحبل السري لطفلي الثاني.
		3- إذا لم أحفظ الخلايا الجذعية لطفلي الأول، فلا يمكن حفظ الخلايا الجذعية لطفلي الثاني.
		4- تقتصر علاجات دم الحبل السري على علاج الأطفال.
		5- يقتصر زرع دم الحبل السري على علاج أمراض الدم فقط.
		6- يمكن التبرع بدم الحبل السري في أي مستشفى.
		7- الأسرة المتوقع تبرعها بدم الحبل السري لديها الوقت حتى الولادة لكي تقرر الموافقة على التبرع.
		8- جمع دم الحبل السري يمكن أن يضر طفلي.
		9- دم الحبل السري هو نفايات طبية لا قيمة لها.
		10- جمع الخلايا الجذعية هو إجراء طبي محفوف بالمخاطر.
		11- حفظ الخلايا الجذعية هي فقط للعائلات التي لديها تاريخ من السرطان.
		12- جمع دم الحبل السري يمكن أن يؤثر على الولادة ويسحب الدم من طفلنا.
		13- دم الحبل السري المحفوظ له "مدة صلاحية محدودة".

شكرا لتعاونك

<u>Authors:</u>

Khaled SAA, Elzembely MM, Soliman AMA, Shawkat N, Rafaat N, Malek MA, Abdelmageed ES 2021.
