# Supplementary material for: Effective and Elaborative Induction Program for Mitigating Myths and Misconceptions Linked to Hematopoietic Stem Cell Transplantation in a Resource Limited Setting
Source: Indian J Hematol Blood Transfus. 2023 Mar 16;39(4):598–609. doi: 10.1007/s12288-023-01634-5 (PMC10542043; doi:10.1007/s12288-023-01634-5)
Supplement: Supplementary file 2 — (PDF 182 KB) [file 12288_2023_1634_MOESM2_ESM.pdf]

Appendix B

A questionnaire to assess common myths and misconceptions about hematopoietic stem cell transplantation

1. What are your major sources of information?

- Hospital educational material.
- Medical personnel.
- Media (TV, Radio, Newspaper, Internet)
- School
- College
- Journal
- Conference
- Others

2. Would you like to receive information about hematopoietic stem cell? transplantation (HSCT). 1-yes 2-No

3. Will you donate stem cells? 1 .Agree 2 .Disagree 3.Not sure 4. Agree only to my family

No	Yes	A. Myths and misconceptions about HSCT
		1.There is only one type of HSCT
		2- A stem cell transplant is a surgical procedure that requires the patient to enterthe operating room and be given general anesthesia
		3-Surgeons perform bone marrow transplantation operations
		4- HSCT is used in treating cancer only
		5- After the recipient is discharged from the hospital, periodic follow-up must be done at regular periods in the hospital
		6- Visiting the recipient is prohibited after the stem cells transfer for a period specified by the doctors

		7- The cure rate is very poor after HSCT
		8- The recipient is kept in the hospital for long periods up to years
		9- The HSCT is a simple procedure with no complications
		10- There is a HSCT center in your area
		**B- Myths and Misconceptions about hematopoietic stem cell donation**
		1-I do not need to be a HSCT donor as long as I am not sick.
		2- Bone marrow donation is done by removing part of the bone and extracting bone marrow from it
		3- Donating bone marrow may be associated with paralysis after donation
		4- Donating bone marrow affects the fertility of men or women
		5- Donating bone marrow makes the patient vulnerable to cancer
		6- Donating HSC is not necessarily from a brother, but is suitable for the father or mother or any relative
		7- Children are not suitable for stem cells donation
		8- Donating stem cells affects the growth of children
		9- A blood transfusion may be given after donating bone marrow
		10 - Donation from a man to a woman is not suitable, or vice versa
		11-The donor may be admitted to the hospital after donation
		12-The age of the donor does not matter.
		13 - Donating HSCT takes a long time, so it is a waste of time.
		14-If you are a woman, the donor must also be a woman.
		15- Surgery is the only way to donate HSC.
		16-Donation is only from relatives.
		17-If I am a woman and the donor is a man, I will have masculine characteristics.
		18- Donating stem cells is dangerous and weakens the donor.
		19 – HSC donation is really painful.
		20-Donation of stem cells includes a long recovery period.
		21-Registration to donate bone marrow requires a blood test.
		22-The bone marrow is taken from the spine.
		23- Donating HSC is very expensive.
		**C: Myths and Misconceptions about umblical cord blood stem cell**

		**preservation**:
		1. If someone in my family needs a cord blood stem cell transplant, it can only be done if I donate cord blood to my baby.
		2. If I preserved the cord blood for my first child, I do not need to preserve the cord blood for my second child.
		3. If I do not preserve my first child's stem cells, my second child's stem cells cannot be saved.
		4. Treatments by cord blood are limited to treating children.
		5. Umbilical cord blood transplantation is restricted to treating blood diseases only.
		6. Umbilical cord blood can be donated in any hospital.
		7. A family expected to donate cord blood has time until birth to decide whetherto donate.
		8. Collecting umbilical cord blood could harm my baby.
		9. Cord blood is worthless medical waste.
		10. Stem cell collection is a risky medical procedure.
		11. -Stem cell preservation is only for families with a history of cancer.
		12. - Umbilical cord blood collection can affect delivery and draw blood from our baby.
		13. Preserved umbilical cord blood has a "limited shelf life."

Thanks for your co-operation

<u>Authors:</u>

Khaled SAA, Elzembely MM, Soliman AMA, Shawkat N, Rafaat N, Malek MA, Abdelmageed ES 2021.
